# Case of Luxation of the Cervical Vertebræ

**Published:** 1830-06

**Authors:** 


### LUXATION OF THE CERVICAL VERTEBRÆ.

### *Case of Luxation of the Cervical Vertebræ.*

### By Dr. Ehrlich.*

Many favorable circumstances most concur, and surgical aid must be very speedily applied, to save the life of a patient who has met with so serious an injury as luxation of the cervical vertebræ. The following case cannot, therefore, be devoid of interest.

A young man, seventeen years old, undertook to carry upon his back a sack of flour, weighing about 160 pounds, to the top of a granary four stories high. To prove his strength and agility, he endeavoured to mount the steps more quickly than his companions, who were laden with a similar burden, and he began to run before them, holding the sack upon his left shoulder. The weather being rainy, the steps were wet and slippy. In his heedless ascent, he struck his load against that of one of his companions, and fell backwards, and still endeavouring to hold the sack, he rolled to the ground. In five minutes after the accident, Dr. Ehrlich arrived. The patient was senseless, in a half-upright position, his knees resting upon the sack. His chest was leaning forwards, but his head, which the weight of the sack had driven backwards with great violence, in rolling over him as he fell down the steps, was resting on the right scapula. On the anterior and left side of the neck, there was a swelling, caused by the second cervical vertebra. The head being completely unfixed, fell from one side to the other by its own weight. The face was of a dark blue colour, resembling that of a person who had been hanged. The eyes projected from the orbits, although the eyelids were closed; the tongue hung out of the mouth, from which flowed an abundant quantity of bloody froth. The lower jaw had dropt, and the veins of the neck were gorged with blood, and appeared like cords. The limbs

* Journal Complement. des Sc. Med.

were motionless, and seemed paralysed. Respiration was very slowly and laboriously performed, with a stertorous noise. The pulse was intermitting, and hardly to be felt. The feces and urine had been discharged involuntarily.

From all these circumstances it was evident that there was pressure upon the spinal marrow, which was easily accounted for from the evident luxation. It was clear that the atlas was thrown on one side of its articulation with the second cervical vertebra, and that the latter bone formed the projection which was visible on the anterior part of the left side of the neck. The transverse ligament of the atlas, and the lateral ligament of the processus dentatus, had not been able to resist the force which had acted upon them; and the capsular ligament of the articular processes, which is loose enough to permit the semi-rotation of the head, had been necessarily lacerated in consequence of the sudden and severe extension it had suffered.

The patient was immediately laid on his back, while one assistant was directed to support the head, and another to grasp the shoulders to make a counter-extension. Dr. E. applied the palm of both hands to the occiput and to the atlas, which was thrown backwards, pressing both his thumbs at the same time on the projected second cervical vertebra. In this manner he endeavoured, while extension was made, to press the atlas forwards, and the second cervical vertebra backwards. After some ineffectual attempts, he succeeded in replacing the vertebræ in their articular surfaces, and at the moment the reduction was effected, a noise was heard by all the assistants. Immediately afterwards the projection in the neck disappeared, and the head was fixed firmly in the body. The tongue was drawn within the mouth, the jaws were closed, and a slight motion was observed in the arms. The patient, however, appeared to be in a profound sleep. The pupils of both eyes were also much dilated. Respiration was now performed much more freely, and without stertor; and during expiration there was no discharge from the mouth: there was, however, cough, with bloody expectoration; the pulse was very quick and irregular. A little wine was very cautiously given, to reestablish the regular and uniform circulation of the blood, but, as the organs of deglutition were still incapable of performing their office, very little was swallowed.

Every precaution was used to keep the head in its natural situation. Compresses were placed round the neck, and a bandage applied to retain it in an upright position. The patient was then very carefully placed in bed, nearly in a sitting posture, his head being supported and rendered motionless by properly arranged cushions. As he was still incapable of swallowing, and remained in a lethargic state, hartshorn was applied to the nose, and ten drops of ether were put into the mouth every half hour. The bandage around the head and neck were constantly moistened with a vinous infusion of aromatic herbs. By these means the patient was somewhat reanimated. He occasionally opened his eyes, expectorated more forcibly when the cough teased him, and could swallow a little better. He soon became restless, and endeavoured to turn himself, but was prevented from so doing by the strict orders of Dr. E. When he could swallow with facility, he was directed to drink freely of a solution of cream of tartar and sugar in water, which acted as a mild aperient. The neck was rubbed with a camphor liniment.

During the night the patient had some tranquil sleep, and coughed but little.

On the following morning he understood what was said to him, and answered any questions, but was perfectly unconscious of what had happened to him; he complained only of pain in the back of the neck, and an uneasiness in the left side of the chest. To diminish the pains in the neck, the vapours from aromatic herbs, boiled in water and saffron, were introduced into the mouth; and, as the patient complained of much distress from the bandage, it was removed, and a plaster then applied round the neck, the head heing fixed so firmly that it could not be moved. As the pain in the neck still increased, and was not relieved by a mild opiate, twelve leeches were applied, which diminished it, and appeased the difficulty in breathing.

It was probable that some small vessel had been ruptured in the lungs, as the bloody expectoration still continued; but Dr. E. was unwilling to take blood by venesection, as he feared that exhaustion might rapidly follow; besides which, he hoped that repose and fresh air would suffice to arrest the slight hemorrhage. There was still a considerable swelling in the neck, extending down to the chest, which arose from extravasated blood, but it dispersed in eight days from the use of a camphor liniment. The spitting of blood also ceased during this time. The patient could now sit upright in bed; he took mild nourishment with a good appetite, and swallowed without difficulty. The cure proceeded rapidly, no other symptom remaining after the frightful accident which had happened, excepting pain in the neck when the head was suddenly moved in a lateral direction.

Morgagni, Duverney,* Mauchant,† Tilesius,‡ and others, are of opinion, from the strength of the ligaments which bind the head to the cervical vertebræ, and those which unite the latter bones with each other, that luxation cannot take place, or at least that it never occurs from hanging. Winslow, J. L. Petit, Ludwig, and Blumenbach, on the contrary, admit, with some restrictions, the possibility of luxation of the cervical vertebræ from various accidents, and especially in persons who have been hanged. It is at all times a very hazardous and imprudent action to raise children by the head. Although the two first cervical vertebræ are very firmly united to the head by ligaments, and the atlas has two ligaments proper to itself, and the head is attached to the vertebral column by the ligamentum nuchæ and numerous muscles, still the ligaments which confine the processus dentatus may be ruptured. In children this process is cartilaginous, and the whole weight of their bodies cannot be safely trusted to so feeble a support.

* DUVERNEY endeavours to prove the impossibility of luxation of the head from the atlas, as well as between the first and second cervical vertebræ.

† MAUCHANT asserts that, in dissecting the bodies of criminals who had been hanged, he never found luxation of the vertebræ. HOFFMAN declares the same.

‡ TILESIUS believes that the death of a person raised up by the head does not generally depend upon luxation of the cervical vertebræ, but upon a displacement of the sphenoid bone.

